# Supplementary material for: The Role of FAS Receptor Methylation in Osteosarcoma Metastasis
Source: Int J Mol Sci. 2023 Jul 29;24(15):12155. doi: 10.3390/ijms241512155 (PMC10418590; doi:10.3390/ijms241512155)
Supplement: Supplementary file 1 [file ijms-24-12155-s001.zip › ijms-2503403-supplementary.pdf]

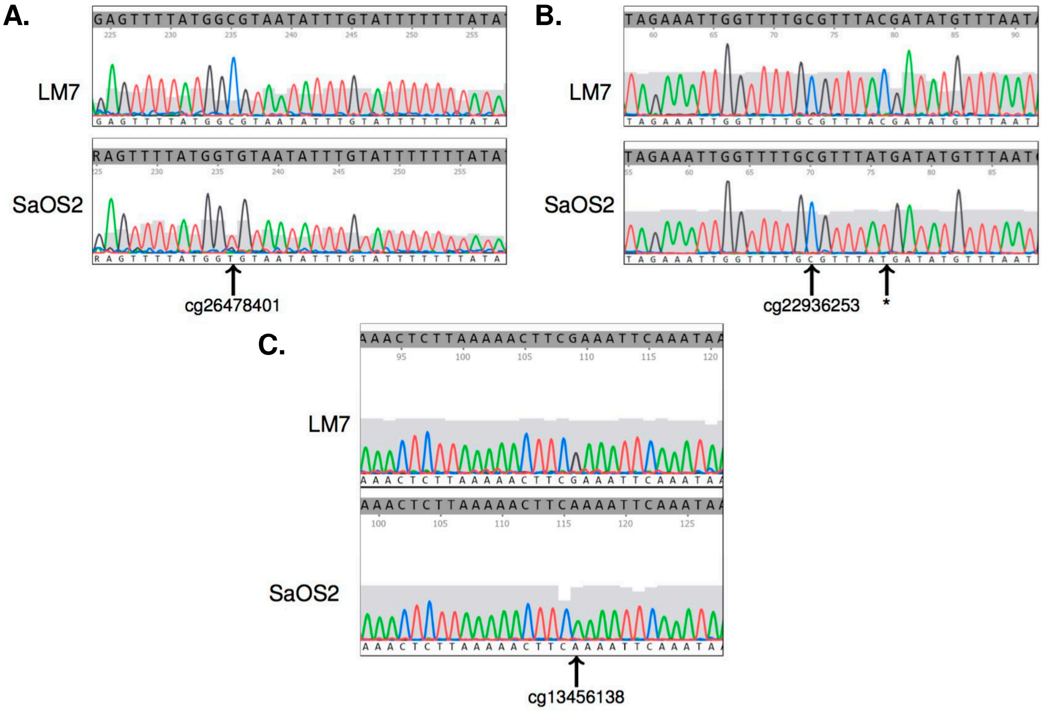

Figure S1: Bisulfite sequencing results from LM7 and SaOS-2 of three HM450 sites. Sequencing results for (a) Site 1, (b) Site 2, and (c) Site 3. * indicates the additional CpG site near Site 2 not on the HM450 array. A/T (green or red) indicates not methylated while C/G (blue, black) indicates methylated.

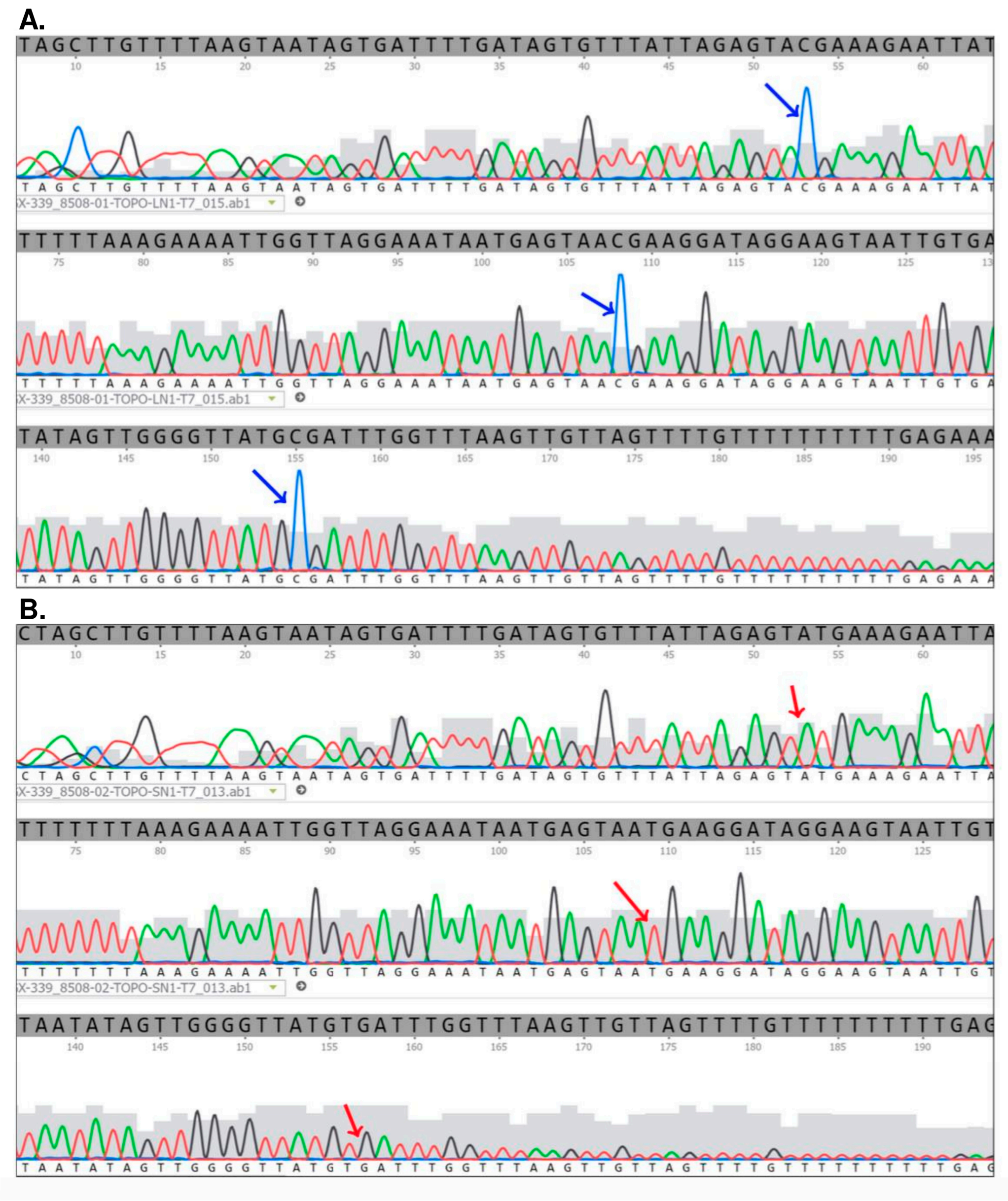

Figure S2: Bisulfite sequencing of three CpG sites upstream of Site 1. Sequencing results of methylation at upstream CpG sites in (a) LM7 and (b) SaOS-2. Consecutive arrows refer to the same sites within LM7 and SaOS2 respectively.

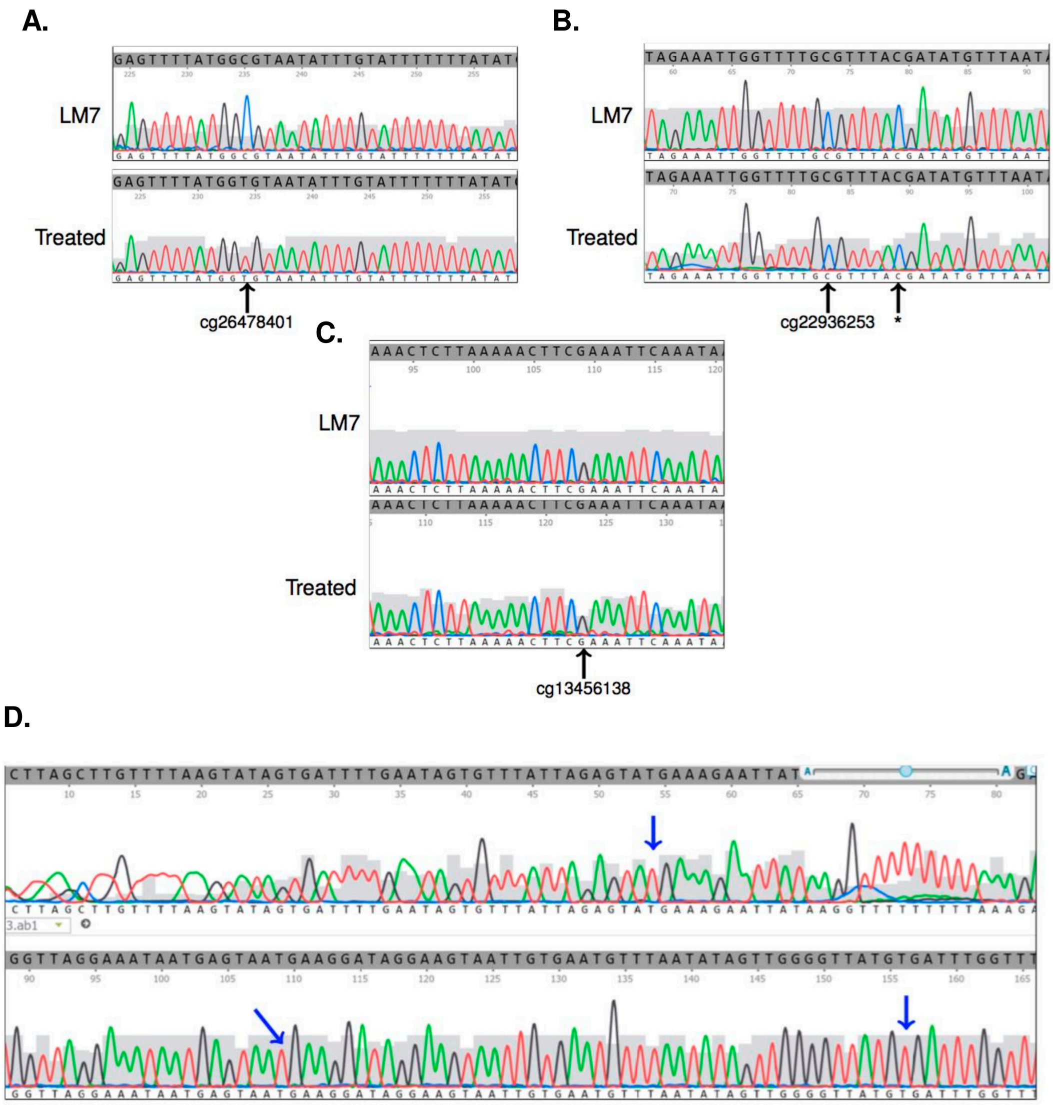

Figure S3: Bisulfite sequencing results after 5-azacytidine treatment. (a) Site 1 is unmethylated after treatment while (b) Site 2 and (c) Site 3 remain methylated. (d) Treated LM7 methylation in three upstream sites from Site are all unmethylated after treatment.

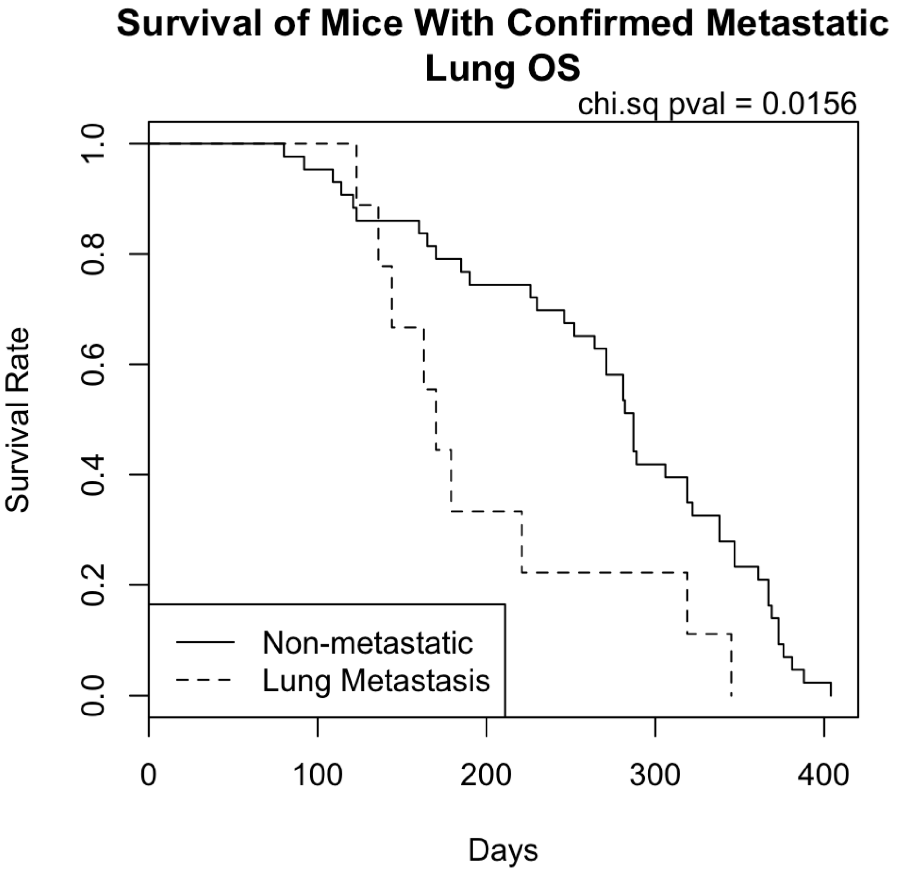

Figure S4: Survival analysis of orthotopically injected mice. Kaplan-Meier curves comparing survival of mice that developed lung metastasis versus mice that did not develop metastasis. Significant differences in survival calculated using a Wald test.

Table S1: Summary of methylation probes in FAS promoter and first intron

TargetID	Mean	S.D.[a]	Rho[b]	p-value	Adj. p-value[c]
cg26478401	0.417	0.236	-0.511	0.000	0.000
cg15774510	0.019	0.015	-0.145	0.181	0.342
cg16257983	0.032	0.035	-0.281	0.009	0.031
cg17120764	0.020	0.014	-0.281	0.009	0.031
cg14538439	0.023	0.005	-0.061	0.577	0.701
cg26461637	0.013	0.003	-0.123	0.258	0.417

cg03387256	0.020	0.004	-0.338	0.002	0.009
cg21565150	0.024	0.007	-0.041	0.708	0.802
cg24417391	0.038	0.006	-0.202	0.062	0.152
cg27008694	0.014	0.003	-0.032	0.770	0.818
cg05358439	0.027	0.007	0.001	0.990	0.990
cg03158499	0.051	0.013	0.114	0.294	0.417
cg19573567	0.059	0.015	0.168	0.122	0.258
cg03520966	0.216	0.037	0.114	0.293	0.417
cg13632959	0.034	0.014	0.100	0.360	0.471
cg22936253	0.726	0.142	-0.597	0.000	0.000
cg03273183	0.954	0.052	0.234	0.031	0.087

[a] Standard deviation
[b] Rho calculated from spearman correlation
[c] Adjusted p-value calculated using Benjamini-Hochberg multiple testing correction
